# Aspartate-β-hydroxylase and hypoxia marker expression in head and neck carcinomas: implications for HPV-associated tumors

**DOI:** 10.1186/s13027-024-00588-1

**Published:** 2024-06-10

**Authors:** Jana Smahelova, Barbora Pokryvkova, Eliska Stovickova, Marek Grega, Ondrej Vencalek, Michal Smahel, Vladimir Koucky, Simona Malerova, Jan Klozar, Ruth Tachezy

**Affiliations:** 1 Department of Genetics and Microbiology, Faculty of Science BIOCEV, Charles University, Prague, Czech Republic; 2 Department of Pathology and Molecular Medicine, Second Faculty of Medicine, Charles University and University Hospital Motol, Prague, Czech Republic; 3 Department of Mathematical Analysis and Applications of Mathematics, Faculty of Science, Palacky University Olomouc, Olomouc, Czech Republic; 4 Department of Otorhinolaryngology and Head and Neck Surgery, First Medical Faculty, Charles University and University Hospital Motol, Prague, Czech Republic

**Keywords:** Hypoxia, Aspartate-β-hydroxylase, Human papillomavirus, Head and neck cancer, Prognosis

## Abstract

**Background:**

A proportion of head and neck carcinomas (HNSCCs) are induced by high-risk human papillomaviruses (HPVs) and are associated with better patient outcomes compared to patients with HNSCCs related to tobacco and alcohol abuse. In the microenvironment of solid tumors, including HNSCCs, oxygen levels are often reduced, and a hypoxic state is induced. This can lead to a poor treatment response and a worse patient prognosis. One of the hypoxia-responsive genes is aspartate-β-hydroxylase (ASPH), whose activity promotes the growth, invasiveness, and metastasis of many types of solid tumors.

**Methods:**

In our study, HNSCC samples were analyzed for the expression of ASPH and selected endogenous hypoxia markers by real-time PCR and/or multiplex fluorescence immunohistochemistry.

**Results:**

Except for the *EPAS1* gene, which had higher mRNA expression in the HPV-negative group of HNSCC (*p* < 0.05), we found no other differences in the expression of the tested genes that were related to HPV status. On the contrary, a statistically significantly higher number of cells producing ASPH (*p* < 0.0001), HIF1A (*p* < 0.0001), GLUT1 (*p* < 0.0001), and MMP13 (*p* < 0.05) proteins were detected in the HPV-positive tumor group than in the HPV-negative sample group. All the evaluated markers, except for MMP9/13, were more abundant in the tumor parenchyma than in the tumor stroma. The Cox proportional hazard models showed that increased numbers of cells with GLUT1 and HIF1A protein expression were positive prognostic markers for overall and disease-specific survival in patients independent of HPV tumor status.

**Conclusion:**

The study examined HNSCC samples and found that elevated ASPH and hypoxia marker proteins, typically associated with poor prognosis, may actually indicate active HPV infection, the strongest prognostic factor in HNSCC patients. In cases where HPV status is uncertain, increased expression of HIF1A and GLUT1 can serve as positive prognostic factors.

**Supplementary Information:**

The online version contains supplementary material available at 10.1186/s13027-024-00588-1.

## Background

Head and neck squamous cell carcinomas (HNSCCs) comprise a heterogeneous group of malignancies with increasing prevalence in developed countries. In addition to cigarette smoking and alcohol consumption, known risk factors include persistent infection with high-risk human papillomaviruses (HPVs), particularly the type of HPV 16. HPV-associated HNSCCs, which are predominantly located in the oropharynx (palatine tonsils, soft palate, and base of the tongue), represent a distinct group of carcinomas with different biological and clinical characteristics [1]. HPV-positive tumors are diagnosed in 5 to 10 years younger patients and have higher involvement of regional lymph nodes. These tumors are associated with greater radiosensitivity and infiltration of immune cells, and patients with these tumors have a better prognosis than HPV-negative patients do [2]. Therefore, the host immune response, the role of tumor-infiltrating lymphocytes, and relevant factors in the tumor microenvironment of HNSCCs are being investigated extensively to improve patient stratification for individualized treatment with the aim of reducing acute and late adverse consequences of treatment [3–5].

In the microenvironment of growing solid tumors, the oxygen concentration is often reduced, and a hypoxic state is induced. The central regulator of the adaptive cellular response to changes in oxygen levels is hypoxia-inducible factor 1 (HIF1), which functions as a transcription factor influencing the expression of many target genes [6]. Products of hypoxia-responsive genes include mainly proteins that influence cell metabolism (e.g., glucose transporter 1 - GLUT1, carbonic anhydrase 9 - CA9, and pyruvate dehydrogenase kinase 1 - PDK1); angiogenesis (e.g., vascular endothelial growth factor - VEGF); and the composition and function of the tumor microenvironment (e.g., matrix metallopeptidases - MMP, prolyl 4-hydroxylase subunit alpha 1 - P4HA1) [7]. The upregulation of these genes allows cells to survive under adverse conditions. Tumor hypoxia has been recognized as one of the biological indicators associated with treatment failure [8]. The clinical utility of hypoxia markers remains still unclear. Overexpression of the central hypoxia factor HIF1A in HNSCCs has been associated with worse patient outcomes [9, 10] and the utility of a hypoxia-responsive gene signature as a tool for patient stratification has been demonstrated [11]. On the other hand, the prognostic value of the 15-gene hypoxia signature was not confirmed in a cohort of patients with oropharyngeal tumors treated with accelerated chemoradiotherapy [12].

Numerous studies have shown that direct and indirect interactions of HPV 16 oncoproteins with the HIF1 factor may also affect the stability and activity of this protein [13–16]. Subsequent changes in cell signaling and metabolism can promote increased cell proliferation and thus HPV production. In addition, other factors, such as smoking and alcohol consumption, may influence the expression of endogenous markers of hypoxia [17]. However, studies on the effects of hypoxia in HNSCC patients in relation to HPV infection and favorable treatment outcomes have been inconclusive [18].

During the malignant transformation of tumor cells, increased expression of aspartate β-hydroxylase (ASPH) has been observed [19]. ASPH belongs to the α-ketoglutarate-dependent dioxygenase family and can serve as an oxygen sensor in cells. It hydroxylates aspartyl and asparaginyl residues mainly in epidermal growth factor-like protein domains, including domains in Notch receptors and ligands, and is thus able to influence numerous cell signaling pathways. ASPH is abundantly expressed in proliferating trophoblast cells, but its activity is low or absent in adult tissues. ASPH overexpression has been shown in many types of carcinomas, including HNSCC, and has been associated with increased tumor cell migration, invasiveness and metastasis, and a significantly negative patient prognosis [20].

This study aimed to compare the expression of ASPH and selected hypoxia markers as potential therapeutic targets at the mRNA and protein levels and determine the prognostic significance of these markers in patients in relation to the viral etiology of HNSCCs.

## Materials and methods

### Study population

Tissue samples from HNSCCs were collected in a previous study at the Department of Otorhinolaryngology and Head and Neck Surgery, First Faculty of Medicine, Charles University, and Motol University Hospital, Prague, in 2017–2020 [21]. The study was approved by the Ethics Committee of Motol University Hospital, Prague, on 22 June 2016. All patients provided signed informed consent and completed a questionnaire on the risk factors for HPV infection and the induction of HNSCC.

### Sample processing and characterization

The samples were processed as described previously [21]. Briefly, after surgical resection, histological examination and pTpN classification (UICC, 8th edition) [22], the tumor was divided by a pathologist into two parts. One part of the tissue sample was fixed in 10% neutral formalin and paraffin-embedded (FFPE) and the other part of the fresh tumor tissue was transported to the laboratory in RPMI medium (Sigma-Aldrich, St. Louis, MO, USA) at 4 °C. The tumor cells together with the tumor-infiltrating cells were immediately isolated from the tumor tissue using the gentleMACS system (Miltenyi Biotec, Auburn, CA, USA). The cell suspension was stored in RNAlater® stabilization solution (Life Technologies, Carlsbad, CA, USA) at -80 °C until further processing. DNA and total RNA were isolated using NucleoSpin® RNA/DNA Buffer Set (Macherey Nagel, Germany) according to the manufacturer’s protocol. The concentration of the nucleic acids was measured using a NanoDrop 2000 Spectrophotometer (Thermo Fisher Scientific, Waltham, MA, USA) and the quality and integrity of the RNA were verified by the Experion™ automated electrophoresis system (Bio-Rad, Hercules, CA, USA) according to the manufacturer’s protocol.

The presence and genotype of HPV were evaluated by PCR with broad spectrum GP5+/6+-5´-bio primers followed by reverse-line blot analysis, and active viral infection was determined by type-specific HPV E6 mRNA detection, both of which were performed as described previously [23, 24].

### Quantification of mRNA expression by quantitative PCR (qPCR)

Total RNA was treated with DNase I (Jena Bioscience, Jena, Germany) and reverse transcribed in a 20-µl reaction mixture using M-MLV Reverse Transcriptase (Promega, Madison, USA) and random hexamers (IDT, Leuven, Belgium), both according to the manufacturer’s instructions.

Gene-specific primers to determine the mRNA expression of ASPH and selected hypoxia markers (HIF1A - HIF1, subunit alpha; SLC2A1 - solute carrier family 2, member 1; P4HA1; VEGFA; EPAS1 - endothelial PAS domain protein 1) were designed and evaluated in our laboratory or by the Gene Core-qPCR and ddPCR Core Facility (BIOCEV, Vestec, Czech Republic) (Table 1). Quantitative PCR was performed with SYBR Green chemistry (Xceed qPCR SG 2× mix Lo-ROX reaction buffer; IAB, Czech Republic) on CFX96™ Touch cycler (Bio-Rad Laboratories, Hercules, CA, USA). Briefly, the 10-µl reaction consisted of 1× Xceed reaction buffer, 400 nM each of forward and reverse primers and 2 µl of 4× diluted cDNA. The amplification consisted of the following steps: initiation of denaturation (3 min at 95 °C), 40 cycles of amplification (10 s at 95 °C and 30 s at 60 °C) with fluorescence reading in the SYBR Green channel and melting curve analysis at the end of the run.

The amplification plots were analyzed using Bio-Rad CFX Maestro software (Bio-Rad Laboratories, Hercules, CA, USA). The relative quantification of mRNA expression was assessed by the ∆∆Ct method using GenEx™ v6 software (TATAA Biocenter, Goteborg, Sweden).

**Table 1:** Primer pairs used for quantification of mRNA expression by qPCR and expected amplicon lengths

Gene	Name		Sequence 5´-3´	Product length (bp)
*ASPH*	Aspartate β-hydroxylase	F	TGGTGATTCCCAAGGAAGGC	110
		R	CTGCCATACCTCGTGCTCAA	
*HIF1A*	Hypoxia inducible factor 1, subunit alpha	F	ACCCATTCCTCACCCATCAAA	134
		R	GTTCTTCTGGCTCATATCCCATC	
*SLC2A1*	Solute carrier family 2, member 1	F	TGGCTACAACACTGGAGTCATC	128
		R	CTGAGAGGGACCAGAGCGTG	
*P4HA1*	Prolyl 4-hydroxylase, subunit alpha 1	F	AGTACATGACCCTGAGACTGGA	84
		R	GGATTTTCATAGCCAGAGAGCC	
*VEGFA*	Vascular endothelial growth factor A	F	GCTGTCTTGGGTGCATTGG	69
		R	GCAGCCTGGGACCACTTG	
*EPAS1*	Endothelial PAS domain protein 1	F	TCAAAGGGCCACAGCGACAA	131
		R	CCAGCTCATAGAACACCTCCGT	
*GUSB*	Glucuronidase beta (reference gene)	F	GAAAATATGTGGTTGGAGAGCTCATT	101
		R	CCGAGTGAAGATCCCCTTTTTA	
*ACTB*	Actin beta (reference gene)	F	CCACGAAACTACCTTCAACTCCA	132
		R	GTGATCTCCTTCTGCATCCTGTC	

F - forward primer, R - reverse primer

### Multispectral immunohistochemistry (mIHC)

From the FFPE samples, 2 μm-thick sections were prepared on SuperFrost® Plus slides (VWR, Belgium). Prior to antibody staining, the FFPE slides were processed as described previously [21]. Heat-induced antigen retrieval (AR) was performed using a microwave in either AR6 (Akoya Biosciences, Menlo Park, CA, USA) or AR9 (Zytomed Systems, Berlin, Germany) buffer, depending on the particular antibody, and the tissue was blocked using Antibody Diluent/Block (Akoya Biosciences) at room temperature (RT) for 10 min. First, we validated the staining pattern of the primary antibodies (ASPH, polyclonal, Novus Biological [Centennial, CO, USA]; HIF1A, clone HA111, Novus Biological; GLUT1, clone EPR3915, Abcam [Cambridge, United Kingdom]; VEGFA, clone EP1176Y, Biocare Medical [Pacheco, CA, USA]; MMP9, clone 5G3, Abcam / Thermo Fisher Scientific [Waltham, MA, USA]; MMP13, clone VIIIA2, Thermo Fisher Scientific; CK - cytokeratin, clone AE1/AE3, Thermo Fisher Scientific). All primary antibodies were diluted in Antibody Diluent/Block except VEGFA, for which Van Gogh Yellow Diluent (Medical Bio Care, Germany) was used. This was followed by incubation with Opal Polymer HRP Ms + Rb as a secondary antibody, then with Opal™ fluorophores, and DAPI counterstain (all from Akoya Biosciences) according to the manufacturer’s instructions. The stained slides were mounted with Fluoromount™ Aqueous Mounting Medium (Sigma-Aldrich, St. Louis, MO, USA). No primary and isotype controls were performed to ensure staining specificity.

Two multiplex panels (A and B) were designed according to the pattern and labelling intensity of the selected antibodies (Table 2) (Additional file 1). Each antibody was assigned to an Opal™ fluorophore with respect to its intensity and possible spectral overlap. After staining with each antibody, the complex of primary and secondary antibodies was removed using AR buffer. Stripping controls were performed to ensure complete removal of this complex.

**Table 2:** Antibody panels for mIHC

Panel	#	Primary antibody (dilution, incubation)	AR buffer (incubation)	Secondary antibody	OPAL (dilution)
A	*1*	VEGFA (1:150, OVN/4°C)	AR9 (30 min)	Opal Polymer HRP Ms + Rb	540 (1:200)
	*2*	HIF1A (1:200, 1 h/RT)	AR6 (60 min)		520 (1:100)
	*3*	ASPH (1:1200, OVN/4°C)	AR6 (15 min)		620 (1:100)
	*4*	Cytokeratin (1:1000, 1 h/RT)	AR6 (15 min)		690 (1:200)
	*5*	DAPI (1:15, 5 min/RT)			
B	*1*	MMP13 (1:200, OVN/4°C)	AR9 (30 min)	Opal Polymer HRP Ms + Rb	650 (1:150)
	*2*	GLUT1 (1:600, 1 h/RT)	AR9 (15 min)		520 (1:150)
	*3*	MMP9 (1:900, OVN/4°C)	AR9 (25 min)		570 (1:150)
	*4*	Cytokeratin (1:1000, 1 h/RT)	AR6 (30 min)		690 (1:200)
	*5*	DAPI (1:15, 5 min/RT)			

AR - antigen retrieval buffer; HRP - horseradish peroxidase, OVN - overnight, RT - room temperature

Five to eight representative regions of interest were randomly selected for each slide, focusing on high-quality tumor tissue, and imaged at 10 × 20 magnification on an Olympus BX43 microscope (Olympus Life Science, USA) using Mantra™ Snap 1.0.0 software (Akoya Biosciences). Images were analyzed using InForm 2.6.0 software (Akoya Biosciences) with pre-built algorithms unique to each panel. The algorithms consisted of linear unmixing of fluorescence spectra and trainable steps of tissue segmentation into the tumor parenchyma (CK-positive), stroma (CK-negative), and background (DAPI-free); step of cell segmentation (nuclei, cytoplasm, and membrane parts); and cell phenotyping. Each step of the algorithm was optimized on a set of different images and subsequently applied to the remaining images. The process of workflow optimization was described previously [5] and was used in this study. Finally, for each sample, three regions of the highest quality were selected from the captured images and then analyzed. For mIHC data analyses, the total number of positive cells was divided by the analyzed area (mm^2^) to obtain comparable, standardized values for the whole area or for the parenchymal and stromal areas separately.

### Statistical analyses

For statistical analyses, the samples were divided into two groups, HPV-positive (HPV+) and HPV-negative (HPV−), based on E6 mRNA HPV positivity, i.e., active HPV infection. The homogeneity of the two groups for various patients’ characteristics was tested using the chi-square test, Fisher exact test or the Mann-Whitney U test. Subsequently, the Mann-Whitney U or Student’s t-test was used to evaluate the differences in relative mRNA expression and the number of positive cells per mm^2^ for each marker tested between tumor groups. Differences between the number of positive cells in the tumor parenchyma and stroma were compared using the paired Wilcoxon test. Pearson and Spearman correlation coefficients were used to assess the correlation between the expression of each marker at the mRNA and protein levels, respectively. The Cox proportional hazard model was used for overall survival (OS) and disease-specific survival (DSS) multivariate analyses. In addition to patient characteristics, tumor clinicopathological characteristics and HPV status, the models included the marker levels from mIHC, separately for the tumor parenchyma, stroma, and total tumor area. The best models were selected according to the Bayesian information criterion (BIC) and Akaike information criterion (AIC). The hazard ratios (HRs) presented below correspond to a difference of 1000 positive cells/mm^2^. The level of significance was considered to be *p* < 0.05 for all the statistical tests. Statistical analyses were performed using GraphPad Prism 8.4.3 (GraphPad Software, Boston, MA, USA) and R version 4.1.2 (R Foundation for Statistical Computing, Vienna, Austria).

## Results

### Study population and characterization of tumor samples

The detailed clinical and pathological characteristics of the study population are summarized in Table 3. A total of 93 HNSCC patients were enrolled. The mean age of the patients was 61.5 years (range 39–89 years), and the majority of the patients were men (70/93; 75.3%). Most of the tumors 73/93 (78.5%) were located in the oropharynx, while 20/93 (21.5%) were located in the oral cavity. For expression analyses at the mRNA level, samples of 67 patients and at the protein level, samples of 93 patients were available.

**Table 3:** Clinical and pathological characteristics of the study population. Samples were stratified into HPV-positive (HPV+), and HPV-negative (HPV−) groups based on the presence of an active HPV infection. Tumor classification is based on the 8th TNM Staging System [22], which includes tumor extent (T), extent of lymph node spread (N), and presence of metastasis (M)

Patients	HPV+ group	HPV− group	Total		*p*-value^c^
	No. (%)	No. (%)	No. (%)		
No. of patients	60 (64.5)	33 (35.5)	93 (100.0)		
Age (years)^##^					0.6615
Mean	61.9	60.8	61.5		
Median	60.5	62.0	61.0		
Range (years)	39–87	46–83	39–87		
Gender^&^					0.0293
Male	50 (83.3)	20 (60.6)	70 (75.3)		
Female	10 (16.7)	13 (39.4)	23 (24.7)		
Tumor location^&^					< 0.0001
Oropharynx	60 (100.0)	13 (39.4)	73 (78.5)		
Oral cavity	0 (0.0)	20 (60.6)	20 (21.5)		
Smoking status^&^					0.0076
Never	27 (45.0)	5 (15.2)	32 (34.4)		
Past/current	33 (55.0)	28 (84.8)	61 (65.6)		
Alcohol consumption^&^					0.0361
Never	23 (38.3)	5 (15.2)	28 (30.1)		
Past/current	37 (61.7)	28 (84.8)	65 (69.9)		
Metastasis^#^					0.0420
Absent	60 (100.0)	30 (90.9)	90 (96.8)		
Present	0 (0.0)	3 (9.1)	3 (3.2)		
p16 status^&^					< 0.0001
Positive	58 (96.7)	0 (0.0)	58 (62.4)		
Negative	2 (3.3)	33 (100.0)	35 (37.6)		
Tumor size (pT)^&^					0.1252
T1	16 (26.7)	11 (33.3)	27 (29.0)		
T2	41 (68.3)	16 (48.5)	57 (61.3)		
T3	2 (3.3)	3 (9.1)	5 (5.4)		
T4	1 (1.7)	3 (9.1)	4 (4.3)		
Nodal status (pN)^&^					0.0001
N0	16 (26.7)	19 (57.6)	35 (37.6)		
N1	37 (61.6)	5 (15.2)	42 (45.2)		
N2	6 (10.0)	4 (12.1)	10 (10.8)		
N3	1 (1.7)	5 (15.2)	6 (6.4)		
Extracapsular spread^&^					0.1413
Absent	39 (65.0)	27 (81.8)	66 (71.0)		
Present	21 (35.0)	6 (18.2)	27 (29.0)		
Tumor stage (pS)^&^					< 0.0001
I	48 (80.1)	8 (24.2)	56 (60.2)		
II	8 (13.3)	8 (24.2)	16 (17.2)		
III	2 (3.3)	4 (12.1)	6 (6.5)		
IV	2 (3.3)	13 (39.4)	15 (16.1)		
Adjuvant treatment^&^					0.0198
Radiotherapy	24 (40.0)	19^a^ (57.6)	43 (46.2)		
Chemoradiotherapy	26 (43.3)	5^b^ (15.2)	31 (33.3)		
Not specified	2 (3.3)	0 (0.0)	2 (2.2)		
No	8 (13.3)	9 (27.3)	17 (18.3)		

^a^ Including one patient who received radiotherapy after a recurrence and one patient who died before radiotherapy

^b^ Including one patient with resection after chemoradiotherapy

^c^*p*-value for homogeneity of the two HNSCC groups for various patient characteristics was tested using the chi-square test^&^, Fisher exact test ^#^ or the Mann-Whitney test^##^

The overall prevalence of HPV DNA in the samples was 61/93 (65.6%). High-risk HPV 16 was detected in 95.1% (58/61) of HPV DNA-positive tumors. Two samples were positive for HPV 35 (2/61; 3.3%), and one was positive for HPV 33 (1/61; 1.6%). None of the oral cavity tumors were HPV DNA positive. Based on the presence of HPV E6 mRNA, we stratified the samples into HPV-associated (HPV+; 60/93 (64.5%)) and HPV-negative tumor groups (HPV−; 33/93 (35.5%)). One sample positive for HPV DNA and negative for HPV E6 mRNA was included in HPV− group.

### The mRNA expression of ASPH and hypoxia markers was similar regardless of the tumor etiology

**Fig. 1:**
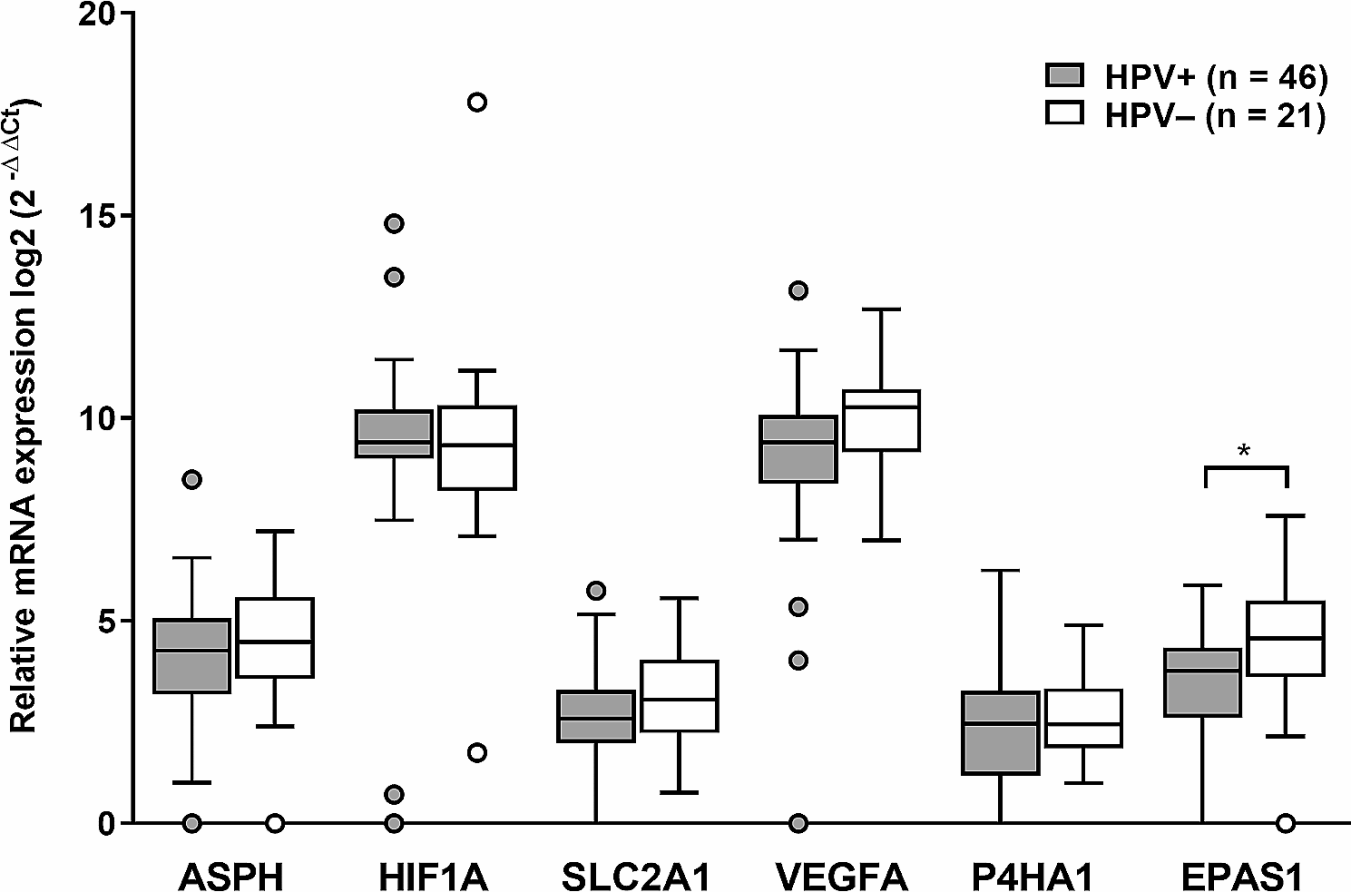
mRNA expression in groups of HPV-positive (HPV+) and HPV-negative (HPV−) HNSCCs. The median values are indicated, the box borders show the upper and lower quartiles, the whiskers show the variability, and outliers are indicated. *p < 0.05

Sixty-seven samples were eligible for mRNA expression testing (46 HPV-positive and 21 HPV-negative). We examined the mRNA expression levels of the selected markers and *ASPH* by reverse transcription followed by qPCR using the relative quantification method. Except for the mRNA expression of *EPAS1* (*p* < 0.05) (Fig. 1), the comparison of the expression of selected markers in HPV-negative and HPV-positive HNSCC groups showed no differences. The statistically significantly positive correlations were found between the expressions of *ASPH* and *P4HA1* (*r* = 0.63, *p* < 0.0001), *ASPH* and *EPAS1* (*r* = 0.57, *p* < 0.0001), *ASPH* and *VEGFA* (*r* = 0.55, *p* < 0.0001), and *ASPH* and *SLC2A1* mRNAs (*r* = 0.31, *p* < 0.01) (data not shown).

### The numbers of cells expressing ASPH and hypoxia markers were markedly different between HPV-positive and HPV-negative tumors, as well as in different tumor compartments

The expression of selected markers at the protein level was determined by the mIHC method, which allows us to reveal the complexity of the tumor microenvironment in a spatial context. In contrast to the lack of differences in the mRNA expression of most of the selected markers, we observed marked differences in the count of cells producing the majority of the evaluated markers between the HPV-positive and HPV-negative groups.

**Fig. 2:**
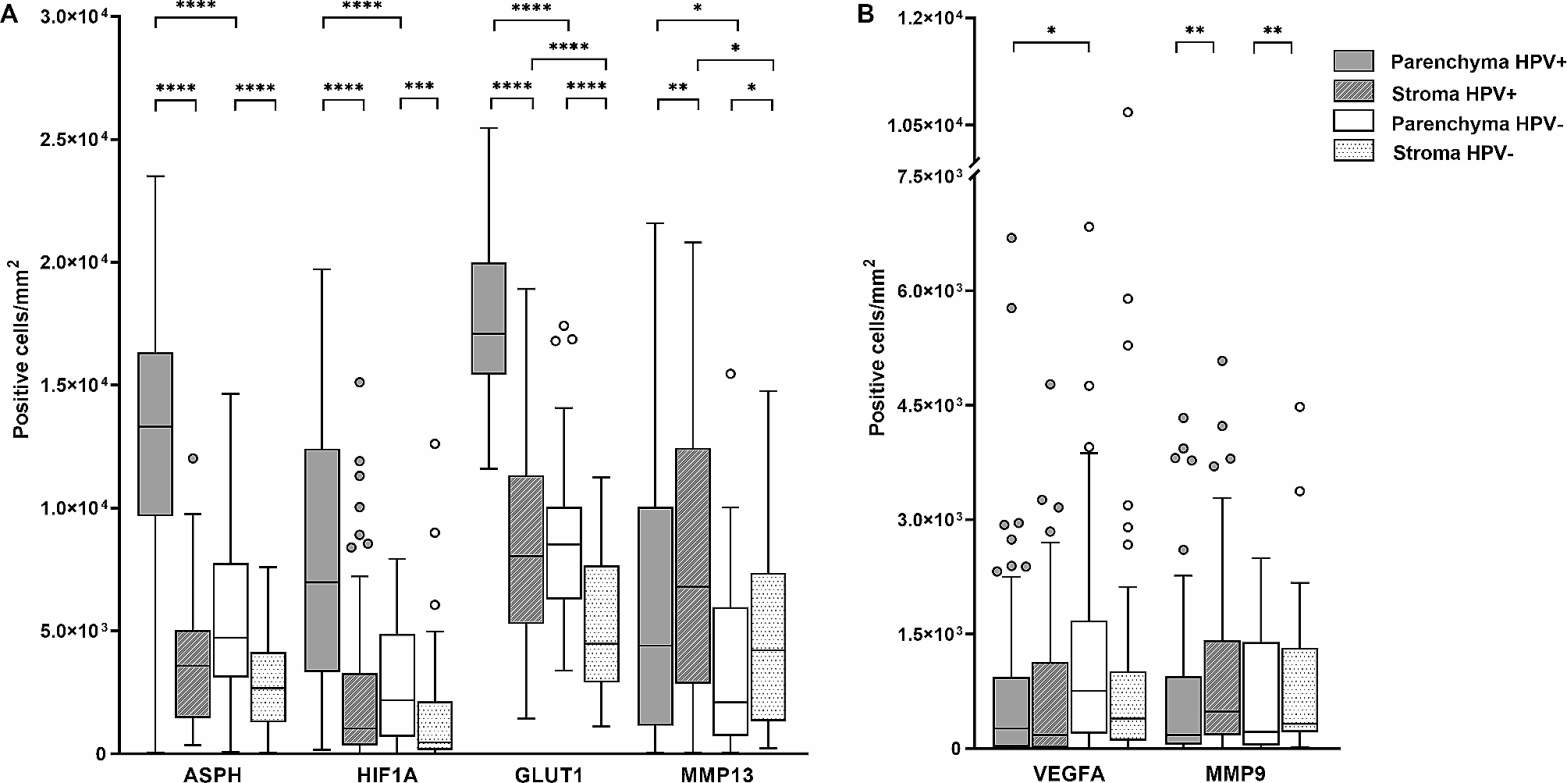
Protein markers detected by mIHC in the parenchyma and stroma of HPV-positive (HPV+) and HPV-negative (HPV−) HNSCCs. Graphs show the number of cells producing (**A**) ASPH, HIF1A, GLUT1, and MMP13, and (**B**) VEGFA and MMP9. The median values are indicated; the box borders show the upper and lower quartiles; the whiskers show the variability, and outliers are indicated. *p < 0.05, **p < 0.01, ***p < 0.001, ****p < 0.0001

When the results were stratified by tumor etiology, cells producing ASPH (*p* < 0.0001), and HIF1A (*p* < 0.0001) were more abundant in the parenchyma of HPV-positive tumors compared to the parenchyma of HPV-negative group. In the HPV-positive tumor group, GLUT1- (*p* < 0.0001) and MMP13-positive cells (*p* < 0.05) were more abundant in both tumor compartments compared to the HPV-negative tumor group (Fig. 2A). On the contrary, the HPV-negative samples showed a higher number of VEGFA-positive cells in the tumor parenchyma than the HPV-positive group (*p* < 0.05). The number of VEGFA-positive cells in the stroma was similar in both groups of patients (Fig. 2B).

Furthermore, when stratifying the results by tumor compartment, the number of parenchymal cells expressing ASPH (*p* < 0.0001), HIF1A (*p* < 0.0001 for HPV-positive; *p* < 0.001 for HPV-negative), and GLUT1 (*p* < 0.0001) was significantly higher compared to the number of positive stromal cells in both HNSCC groups. In contrast, the number of MMP9- (*p* < 0.01) and MMP13-positive cells (*p* < 0.01 for HPV-positive; *p* < 0.05 for HPV-negative) was significantly higher in the stroma of both tumor groups (Fig. 2).

In addition to the above analysis, we compared the positive cells counts in the total tumor area (parenchyma and stroma) in both tumor groups. Significantly higher numbers of cells expressing ASPH (*p* < 0.0001) and the hypoxia markers – HIF1A (*p* < 0.0001), GLUT1 (*p* < 0.0001), and MMP13 (*p* < 0.05) – were detected in HPV-positive than in HPV-negative tumors (Additional file 2A). There were also marked differences in the positive cell counts when comparing different tumor compartments without HPV status stratification (Additional file 2B). The numbers of cells expressing ASPH (*p* < 0.0001), HIF1A (*p* < 0.0001), and GLUT1 (*p* < 0.0001) were significantly higher in the tumor parenchyma compared to the stroma, while cells expressing MMP13 (*p* < 0.001) and MMP9 (*p* < 0.0001) were more abundant in the stroma of HNSCCs (Additional file 2B). These findings are similar to the analysis involving HPV status (Fig. 2).

### Several hypoxia markers were strongly correlated

**Fig. 3:**
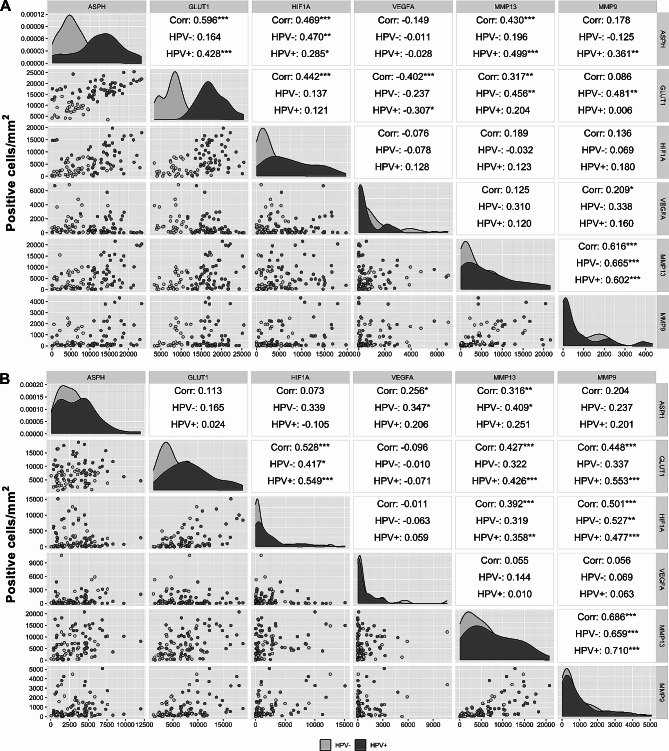
Correlation of the number of cells producing ASPH and hypoxia markers in the parenchyma (**A**) and the stroma (**B**) of HNSCCs. The viral etiology of the tumors is indicated by the color. Spearman correlation coefficient is determined for the whole cohort of HNSCCs (Corr), and separately according to viral etiology of tumors (HPV+, HPV−). *p < 0.05, **p < 0.01, ***p < 0.001

We observed strong positive correlation in the parenchymal and stromal count of cells expressing MMP13 and MMP9 in both group of samples independent their HPV status (HPV-positive tumors: parenchyma rS = 0.602, *p* < 0.001, stroma rS = 0.710, *p* < 0.001; HPV-negative tumors: parenchyma rS = 0.665, *p* < 0.001, stroma rS = 0.659, *p* < 0.001). Additionally, a significantly positive correlations were observed in the abundance of parenchymal cells producing ASPH and GLUT1 (rS = 0.428, *p* < 0.001), ASPH and HIF1A (rS = 0.285, *p* < 0.05), ASPH and MMP13 (rS = 0.499, *p* < 0.001), and ASPH and MMP9 (rS = 0.361, *p* < 0.01) in the HPV-positive tumor group. In the parenchyma of HPV-negative cohort of tumors, the positive correlation was detected between ASPH and HIF1A (rS = 0.470, *p* < 0.01), GLUT1 and MMP13 (rS = 0.456, *p* < 0.01), and GLUT1 and MMP9 (rS = 0.481, *p* < 0.01) numbers of cells. A significantly negative correlation was observed between VEGFA- and GLUT1-positivity (rS = − 0.307, *p* < 0.05) in the parenchyma of HPV-positive tumors (Fig. 3).

### Associations of hypoxia markers with patient outcomes

**Fig. 4:**
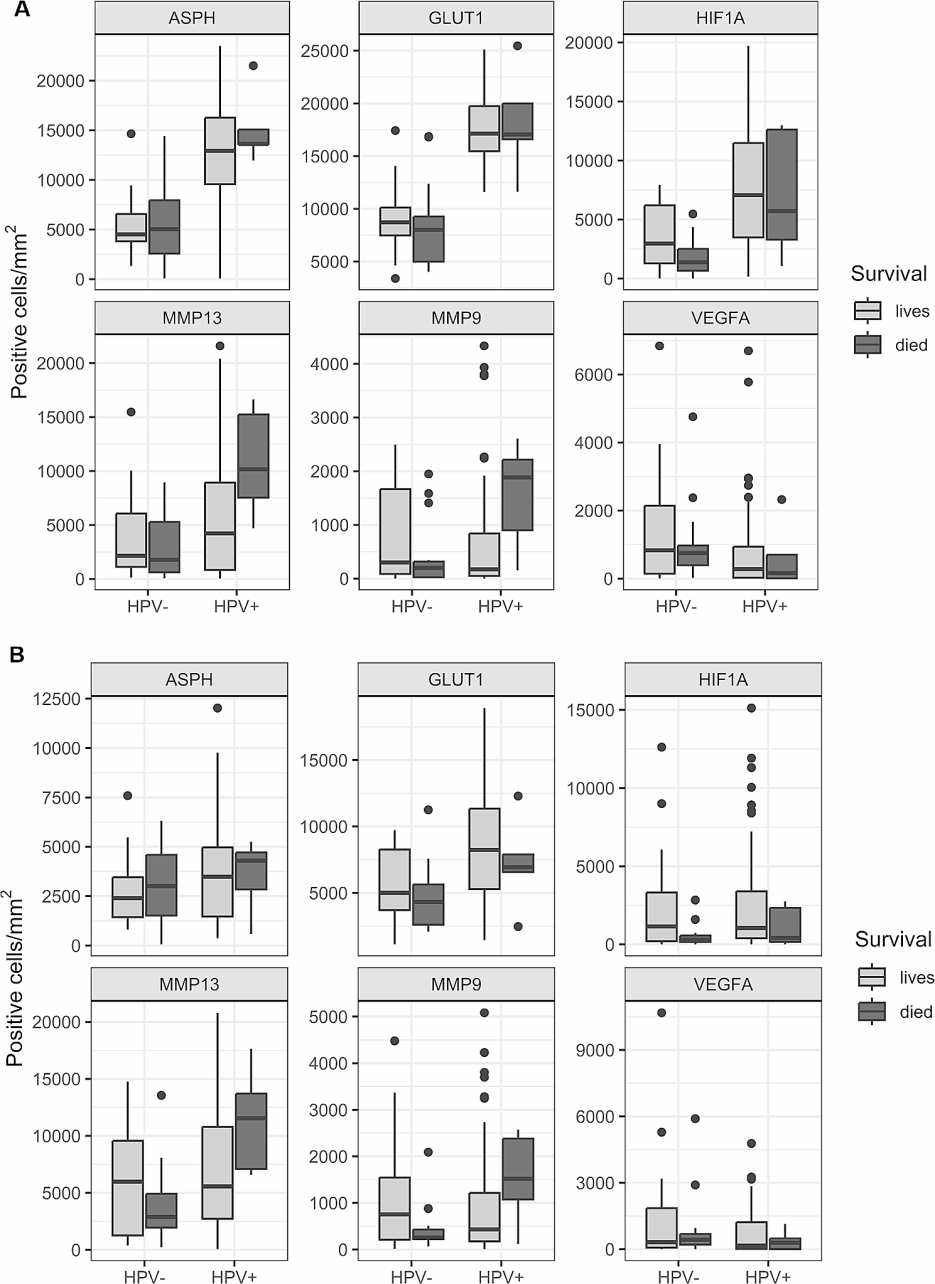
Association of ASPH and hypoxia markers in the parenchyma (**A**) and the stroma (**B**) of HNSCCs according to HPV etiology of tumors and patient outcomes

In both etiologically distinct groups of tumors, we compared the number of parenchymal and stromal cells positive for the analyzed markers between patients who died and those who were alive at follow-up. The results showed that the number of parenchymal cells producing HIF1A was higher in patients who were alive, but the difference was statistically significant only in the HPV-negative group (*p* < 0.05). In addition, MMP13 and MMP9 counts of positive parenchymal cells were significantly higher in the patients who died (*p* < 0.05 and *p* < 0.0001, respectively), but these differences were observed only in the HPV-positive group of subjects (Fig. 4).

### Analyses of overall and disease-specific survival showed the prognostic significance of GLUT1- and HIF1A-positive cell counts

The mIHC results were used in multivariate Cox proportional hazards models to determine the impact of ASPH and hypoxia marker expression on patient prognosis. The best models selected by the BIC for initial evaluation included HPV status and age for both OS (HPV, HR = 0.125, *p* = 0.001; age, HR = 1.061, *p* = 0.020) and DSS (HPV, HR = 0.141, *p* = 0.002; age, HR = 1.075, *p* = 0.019). Each of the IHC markers was evaluated in these models in the whole tumor area and separately in the tumor parenchyma and stroma. None of the selected IHC markers were significantly prognostic for OS or DSS. The best model for improved OS included younger age, positive HPV status, and higher numbers of cells producing HIF1A but without statistical significance (*p* = 0.060) (Additional file 3). We also tested Cox models in which the HPV status, the strongest prognostic factor, was omitted. According to these models, the numbers of GLUT1-positive cells in the whole tumor area and also separately in the tumor parenchyma and stroma were prognostic factors for both OS (HR = 0.802, *p* < 0.0001; HR = 0.852, *p* < 0.0001; HR = 0.814, *p* = 0.007, respectively) and DSS (HR = 0.814, *p* = 0.005; HR = 0.862, *p* = 0.009; HR = 0.842, *p* = 0.032, respectively). Furthermore, the numbers of cells producing HIF1A in the whole tumor area (HR = 0.729, *p* = 0.003), tumor parenchyma (HR = 0.829, *p* = 0.008), and stroma (HR = 0.616, *p* = 0.027) were prognostic factors for OS. The number HIF1A-positive cells in the whole tumor area (HR = 0.773, *p* = 0.021) was also prognostic for DSS. These results also support our observation of a close relationship between HPV status and the numbers of cells producing GLUT1 and HIF1A proteins in HNSCCs (Fig. 3).

## Discussion

We investigated the expression of ASPH and selected hypoxia markers in relation to the viral etiology of HNSCC and patient survival. The abundance of cells producing ASPH and the hypoxia markers GLUT1, HIF1A, and MMP13 were statistically significantly higher in the group of HNSCC samples associated with HPV infection, while almost no differences were detected at the mRNA level. We demonstrated a strong correlation between high number of GLUT1-positive cells and HPV positivity and consequently observed an association of the higher numbers of GLUT1- and/or HIF1A-producing cells with improved OS or DSS in HNSCC patients in Cox hazard models where the HPV status was omitted.

To our knowledge, this is the first study to evaluate ASPH expression in clinical samples of HNSCC patients in relation to the viral status of tumors. High expression of the *ASPH* gene has been identified as a component of the oxygen-sensing gene signature associated with poor prognosis in patients with several types of carcinomas, including HNSCC [25]. However, the viral etiology of HNSCC was not considered in that study, and tumors of different anatomical locations of head and neck were combined into one group. In our analyses, we found a significantly increased abundance of cells producing ASPH at the protein level in HPV-positive samples. High ASPH production has been associated with more aggressive tumor behavior and metastasis in many types of solid tumors [20]. However, this marker was not recognized as an independent risk factor for patient survival in our study.

Factors that contribute to ASPH upregulation include growth factors that also activate the phosphatidylinositol-3-kinase/protein kinase B (PI3K/Akt) signaling pathway [26]. Since activation of PI3K/Akt signaling is essential for HPV-induced carcinogenesis [27], it could be responsible for the increased ASPH synthesis in HPV-positive HNSCCs found in our study. Increased ASPH expression may also be mediated by HIF1A activity, as has been shown in neuronal cells [28].

In our group of clinical samples, where HPV-positive tumors predominated, we found no differences in the tested gene expression patterns that were associated with HPV status, except for the *EPAS1* gene. In contrast to those at the mRNA level, the numbers of cells producing ASPH and hypoxia markers at the protein level were significantly different between the non-HPV and HPV-positive tumor groups. Expression of genes at the mRNA level may differ from that at the protein synthesis level, as has been repeatedly demonstrated [29, 30]. In addition, the selective translation of mRNAs, which are essential for cell survival, may play a role in hypoxia [31].

Finally, the influence of HPV infection on cellular regulation cannot be excluded. The significant relationship between HR HPV 16 infection and HIF1A expression has been demonstrated in various cancers, including cervical, lung cancer and HNSCC [16, 32–34]. The central hypoxia factor HIF1A can also be stabilized by direct interaction with the HPV 16 E6 oncoprotein [13] and accumulated under normoxic conditions [35]. In the study by Rodolico et al. [36], an oxygen-independent positive association was observed between HIF1A and HPV 16 E7 immunoreactivity in oral squamous cell carcinoma. The significantly elevated numbers of cells producing HIF1A protein observed in HPV-associated tumors in our study may therefore be a consequence of HPV infection rather than a manifestation of actual tumor hypoxia.

Patients with HPV-positive HNSCCs have a better overall prognosis and the tumors are more radiosensitive [2]. According to the Cox proportional hazard models, a positive HPV status and younger patient age improved both OS and DSS in our study, which is in agreement with the conclusions of our study and other previous studies [5, 37, 38]. In these models, we did not observe a significant relationship between the number of cells producing ASPH or the hypoxia markers and the prognosis of HNSCC patients. On the contrary, in the models where the HPV status was omitted, we found that higher levels of cells producing HIF1A and GLUT1, which may reflect the HPV status of tumors, were associated with improved OS and DSS in HNSCC patients. As mentioned above, tumor hypoxia may be connected with treatment failure and poor patient prognosis [8]. In a systematic review, Gong et al. showed a significant association between HIF overexpression and increased mortality risk in HNSCC patients [39]. However, in detailed subgroup analyses, they observed a significantly increased mortality risk associated with HIF1A overexpression in studies from Asia, but not in European patients. In addition, the prognostic value of increased HIF1A expression varied in different HNSCC disease subgroups. Consistent with our study, two studies included in the previous meta-analysis demonstrated significantly better OS and also disease-free survival in HNSCC patients with increased HIF1A protein levels [40, 41].

Alterations in cellular metabolism and adaptation to increased energy consumption by cancer cells have been shown in a variety of tumors [7]. This is associated with increased glucose transport and elevated activity of glycolytic enzymes. Among the glucose transporters, GLUT1, the product of the *SLC2A1* gene, has received the most attention from researchers, and its upregulation has been recognized as a negative prognostic factor in many tumor types. Two meta-analyses have reported an adverse impact of GLUT1 overexpression in solid tumors, including oral squamous cell carcinoma, on patient outcomes [42, 43]. However, these studies did not consider HPV tumor status, and oral cancers are rarely associated with HPV infection. In our study, significantly higher numbers of GLUT1-positive cells were found in the group of HPV-positive HNSCCs. An active HPV infection may again play a role, as HPV proteins stimulate cellular signaling pathways that promote glucose uptake and glycolysis [44]. In HPV-positive lung carcinomas, the studies by Fan et al. [45] and Tang et al. [33] showed that GLUT1 expression could be affected by the activities of HPV 16 E6/E7 oncoproteins.

In our study, multiplex IHC analysis allowed us to further assess the spatial distribution of ASPH and other hypoxia markers in the tumor microenvironment. While ASPH-, HIF1A-, and GLUT1-positive cells were more abundant in the tumor parenchyma, MMP9/13-expressing cells were more abundant in the tumor stroma. As MMPs are also known to be products of stromal fibroblasts, lymphocytes, granulocytes, and activated macrophages, this may reflect the higher infiltration of these cells in the stroma compared to the parenchyma of HNSCCs, which has been described by our group in the previous study [21] as well as by others [46, 47]. In addition, the E7 oncoprotein of high-risk HPV has been recognized as a factor directly contributing to the increased expression of MMPs, including MMP9 [48]. Therefore, HPV-infected keratinocytes may exhibit more aggressive behavior than those not infected with the virus. Our study showed significantly increased numbers of cells producing MMP13 but not MMP9 in patients with HPV-positive tumors, and in these patients, the increased level of MMP9/13-positive cells suggested a worse OS compared to the patients with lower abundance of MMP9/13-positive cells.

Among other hypoxia markers, the expression of VEGFA, the key proangiogenic factor in the tumor microenvironment that promotes tumor neovascularization, was evaluated. In addition to HIF1A, VEGFA expression is also supported by the HPV E6 and E7 oncoproteins, suggesting a possible difference in VEGFA levels in tumors of different etiologies [49]. In our study, similar levels of VEGFA mRNA were observed in both groups. In contrast to our findings, higher VEGFA mRNA levels have been reported in HPV-positive samples compared to HPV-negative ones [50] and, inconsistently, the overexpression of *VEGFA* in p16-negative samples corresponding to HPV-negative tumors has been reported [51]. In our study, higher numbers of VEGFA-positive cells were detected in the parenchyma and the whole tumor area of HPV-negative samples compared to the HPV-positive samples. These data confirm the results of our retrospective study in HNSCC patients (unpublished data) and are consistent with the findings of Baruah et al. [52], who observed higher VEGFA levels in the parenchyma of p16-negative HNSCC patients. Several studies have observed the same level of the VEGFA protein in tumors regardless of HPV status [37, 50, 53]. These discrepancies may be due to the different methodologies used for VEGFA quantification – the very precise method used in our study (positive cell count/mm^2^) and the less precise method used in other studies (weak vs. strong expression).

We did not observe any effect of the VEGFA-positive cell amount on prognosis, which is in line with the findings of some studies [37, 51] while worse OS or DSS has been found in HNSCC patients with increased VEGF protein level by others [54, 55]. However, in these studies, HPV status was not included in the survival analyses, which may have affected the results of these studies.

This study has several limitations. As the number of intact samples usable for reverse transcription and qPCR analyses was relatively low, the evaluated differences might not be statistically significant. Additionally, investigating the expression levels of viral oncoproteins, which may influence the protein level of hypoxia markers and their activity, would be interesting. The heterogeneity of the treatment modalities may also influence the prognostic impact of the variables analyzed in our study, but our cohort was relatively homogeneous because all patients were treated with surgery, and the majority with subsequent radiotherapy or chemoradiotherapy. Lastly, the majority of patients in our study had tumor localized in the oropharynx and more tumors were HPV-positive.

## Conclusions

The examination of HNSCC samples suggested that elevated ASPH and hypoxia marker protein levels, typically indicative of unfavorable prognosis, may reflect the presence of active HPV infection, the strongest prognostic factor in HNSCC patients, rather than tumor hypoxia itself. Even in cases where HPV status is uncertain, increased expression of HIF1A and GLUT1 may serve as positive prognostic factors for HNSCC patients. It should be considered when individualizing therapy for patients with HNSCC of different etiologies.

### Electronic supplementary material

Below is the link to the electronic supplementary material.

**Additional file 1. Figure S1**. Representative multispectral IHC staining of FFPE HNSCC tissue, 20× magnification. (**A**) Panel **A**: Staining of GLUT1 (magenta), MMP9 (green), MMP13 (orange), pan cytokeratin AE1/AE3 (CK, red), and DAPI (blue) antibodies. (**B**) Panel **B**: Staining of HIF1A (magenta), VEGFA (green), ASPH (yellow), CK (red), and DAPI (blue) antibodies.

**Additional file 2. Figure S2**. ASPH and other hypoxia markers detected by the mIHC in the groups of HPV-positive (HPV+) and HPV-negative (HPV−) tumors (**A**), and in the parenchyma and stroma (**B**) of HNSCCs. The median value is indicated; the box borders show the upper and lower quartiles, the whiskers show the variability, and outliers are indicated. *p < 0.05, ** *p* < 0.01, *** *p* < 0.001, **** *p* < 0.0001.

**Additional file 3. Table S1**. Hazard ratio (HR) values for hypoxia markers influencing overall survival (OS) and disease-specific survival (DSS).

## Data Availability

The dataset supporting the conclusions of this article is available in the Zenodo repository; doi: 10.5281/zenodo.10405843.

## Abbreviations

AR: Antigen retrieval
ASPH: Aspartate–β–hydroxylase
CA9: Carbonic anhydrase 9
CK: Cytokeratin
DAPI: 4′,6–diamidino–2–phenylindole
DSS: Disease–specific survival
EPAS1: Endothelial PAS domain protein 1 (synonym HIF2A)
FFPE: Formalin–fixed paraffin–embedded
GLUT1: Glucose transporter protein type 1
HIF1: Hypoxia inducible factor 1
HNSCC: Head and neck squamous cell carcinoma
HPV: Human papillomavirus
HR: Hazard ratio
HRP: Horseradish peroxidase
mIHC: Multispectral immunohistochemistry
MMP9: Matrix metallopeptidase 9
MMP13: Matrix metallopeptidase 13
OS: Overall survival
OVN: Overnight
P4HA1: Prolyl 4–hydroxylase subunit alpha 1
PDK1: Pyruvate dehydrogenase kinase 1
qPCR: Quantitative polymerase chain reaction
RT: Room temperature
SLC2A1: Solute carrier family 2, member 1
VEGFA: Vascular endothelial growth factor A

## References

[1] Wittekindt C, Wagner S, Sharma SJ, Würdemann N, Knuth J, Reder H (2018). HPV – a different view on Head and Neck Cancer. Laryngorhinootologie.

[2] Göttgens E-L, Ostheimer C, Span PN, Bussink J, Hammond EM (2019). HPV, hypoxia and radiation response in head and neck cancer. Br J Radiol.

[3] Almangush A, Jouhi L, Atula T, Haglund C, Mäkitie AA, Hagström J (2022). Tumour-infiltrating lymphocytes in oropharyngeal cancer: a validation study according to the criteria of the International Immuno-Oncology Biomarker Working Group. Br J Cancer.

[4] Bisheshar SK, van der Kamp MF, de Ruiter EJ, Ruiter LN, van der Vegt B, Breimer GE (2022). The prognostic role of tumor associated macrophages in squamous cell carcinoma of the head and neck: a systematic review and meta-analysis. Oral Oncol.

[5] Pokrývková B, Grega M, Klozar J, Vencálek O, Nunvář J, Tachezy R (2022). PD1 + CD8 + cells are an independent prognostic marker in patients with Head and Neck Cancer. Biomedicines.

[6] Semenza GL (2001). HIF-1 and mechanisms of hypoxia sensing. Curr Opin Cell Biol.

[7] Maxwell PH, Pugh CW, Ratcliffe PJ (2001). Activation of the HIF pathway in cancer. Curr Opin Genet Dev.

[8] Multhoff G, Vaupel P (2020). Hypoxia compromises Anti-cancer Immune responses. Adv Exp Med Biol.

[9] Swartz JE, Pothen AJ, van Kempen PMW, Stegeman I, Formsma FK, Cann EMV (2016). Poor prognosis in human papillomavirus-positive oropharyngeal squamous cell carcinomas that overexpress hypoxia inducible factor-1α. Head Neck.

[10] Hong A, Zhang M, Veillard A-S, Jahanbani J, Lee CS, Jones D (2013). The prognostic significance of hypoxia inducing factor 1-α in oropharyngeal cancer in relation to human papillomavirus status. Oral Oncol.

[11] Toustrup K, Sørensen BS, Alsner J, Overgaard J (2012). Hypoxia Gene Expression Signatures as prognostic and predictive markers in Head and Neck Radiotherapy. Semin Radiat Oncol.

[12] Deschuymer S, Sørensen BS, Dok R, Laenen A, Hauben E, Overgaard J (2020). Prognostic value of a 15-gene hypoxia classifier in oropharyngeal cancer treated with accelerated chemoradiotherapy. Strahlenther Onkol.

[13] Nakamura M, Bodily JM, Beglin M, Kyo S, Inoue M, Laimins LA (2009). Hypoxia-specific stabilization of HIF-1alpha by human papillomaviruses. Virology.

[14] Bodily JM, Mehta KPM, Laimins LA (2011). Human papillomavirus E7 enhances hypoxia-inducible factor 1-mediated transcription by inhibiting binding of histone deacetylases. Cancer Res.

[15] Guo Y, Meng X, Ma J, Zheng Y, Wang Q, Wang Y (2014). Human papillomavirus 16 E6 contributes HIF-1α induced Warburg effect by attenuating the VHL-HIF-1α interaction. Int J Mol Sci.

[16] Knuth J, Sharma SJ, Würdemann N, Holler C, Garvalov BK, Acker T (2017). Hypoxia-inducible factor-1α activation in HPV-positive head and neck squamous cell carcinoma cell lines. Oncotarget.

[17] Bredell MG, Ernst J, El-Kochairi I, Dahlem Y, Ikenberg K, Schumann DM (2016). Current relevance of hypoxia in head and neck cancer. Oncotarget.

[18] Wegge M, Dok R, Nuyts S (2021). Hypoxia and its influence on Radiotherapy response of HPV-Positive and HPV-Negative Head and Neck Cancer. Cancers (Basel).

[19] Ince N, Monte SM, de la, Wands JR (2000). Overexpression of human aspartyl (asparaginyl) β-Hydroxylase is Associated with Malignant Transformation. Cancer Res.

[20] Kanwal M, Smahel M, Olsen M, Smahelova J, Tachezy R (2020). Aspartate β-hydroxylase as a target for cancer therapy. J Exp Clin Cancer Res.

[21] Pokrývková B, Šmahelová J, Dalewská N, Grega M, Vencálek O, Šmahel M (2021). ARG1 mRNA level is a promising prognostic marker in Head and Neck squamous cell carcinomas. Diagnostics (Basel).

[22] Amin MB, Greene FL, Edge SB, Compton CC, Gershenwald JE, Brookland RK (2017). The Eighth Edition AJCC Cancer staging Manual: continuing to build a bridge from a population-based to a more personalized approach to cancer staging. CA Cancer J Clin.

[23] Tachezy R, Smahelova J, Salakova M, Arbyn M, Rob L, Skapa P (2011). Human papillomavirus genotype distribution in Czech women and men with diseases etiologically linked to HPV. PLoS ONE.

[24] Rotnáglová E, Tachezy R, Saláková M, Procházka B, Košl’abová E, Veselá E (2011). HPV involvement in tonsillar cancer: prognostic significance and clinically relevant markers. Int J Cancer.

[25] Chang WH, Forde D, Lai AG (2019). Dual prognostic role of 2-oxoglutarate-dependent oxygenases in ten cancer types: implications for cell cycle regulation and cell adhesion maintenance. Cancer Commun (Lond).

[26] Hou G, Xu B, Bi Y, Wu C, Ru B, Sun B (2018). Recent advances in research on aspartate β-hydroxylase (ASPH) in pancreatic cancer: a brief update. Bosn J Basic Med Sci.

[27] Henken FE, Banerjee NS, Snijders PJF, Meijer CJLM, De-Castro Arce J, Rösl F (2011). PIK3CA-mediated PI3-kinase signalling is essential for HPV-induced transformation in vitro. Mol Cancer.

[28] Lawton M, Tong M, Gundogan F, Wands JR, de la Monte SM (2010). Aspartyl-(asparaginyl) β-Hydroxylase, Hypoxia-Inducible Factor-1α and notch cross-talk in regulating neuronal motility. Oxid Med Cell Longev.

[29] Vogel C, Marcotte EM (2012). Insights into the regulation of protein abundance from proteomic and transcriptomic analyses. Nat Rev Genet.

[30] Edfors F, Danielsson F, Hallström BM, Käll L, Lundberg E, Pontén F (2016). Gene-specific correlation of RNA and protein levels in human cells and tissues. Mol Syst Biol.

[31] Chee NT, Lohse I, Brothers SP (2019). mRNA-to-protein translation in hypoxia. Mol Cancer.

[32] Bachtiary B, Schindl M, Pötter R, Dreier B, Knocke TH, Hainfellner JA (2003). Overexpression of Hypoxia-inducible factor 1α indicates diminished response to Radiotherapy and unfavorable prognosis in patients receiving Radical Radiotherapy for cervical Cancer1. Clin Cancer Res.

[33] Tang J-Y, Li D-Y, He L, Qiu X-S, Wang E-H, Wu G-P. HPV 16 E6/E7 promote the glucose uptake of GLUT1 in Lung Cancer through downregulation of TXNIP due to inhibition of PTEN Phosphorylation. Front Oncol. 2020;10. 10.3389/fonc.2020.559543 PMC7689016 33282728

[34] Priego-Hernández VD, Arizmendi-Izazaga A, Soto-Flores DG, Santiago-Ramón N, Feria-Valadez MD, Navarro-Tito N (2023). Expression of HIF-1α and genes involved in glucose metabolism is increased in Cervical Cancer and HPV-16-Positive cell lines. Pathogens.

[35] Kappler M, Pabst U, Rot S, Taubert H, Wichmann H, Schubert J (2017). Normoxic accumulation of HIF1α is associated with glutaminolysis. Clin Oral Investig.

[36] Rodolico V, Arancio W, Amato MC, Aragona F, Cappello F, Di Fede O (2011). Hypoxia inducible factor-1 alpha expression is increased in infected positive HPV16 DNA oral squamous cell carcinoma and positively associated with HPV16 E7 oncoprotein. Infect Agent Cancer.

[37] Fei J, Hong A, Dobbins TA, Jones D, Soon Lee C, Loo C (2009). Prognostic significance of vascular endothelial growth factor in squamous cell carcinomas of the Tonsil in relation to human papillomavirus status and epidermal growth factor receptor. Ann Surg Oncol.

[38] Ang KK, Sturgis EM (2012). Human papillomavirus as a marker of the natural history and response to therapy of head and neck squamous cell carcinoma. Semin Radiat Oncol.

[39] Gong L, Zhang W, Zhou J, Lu J, Xiong H, Shi X (2013). Prognostic value of HIFs expression in head and neck cancer: a systematic review. PLoS ONE.

[40] Beasley NJP, Leek R, Alam M, Turley H, Cox GJ, Gatter K (2002). Hypoxia-inducible factors HIF-1alpha and HIF-2alpha in head and neck cancer: relationship to tumor biology and treatment outcome in surgically resected patients. Cancer Res.

[41] Fillies T, Werkmeister R, van Diest PJ, Brandt B, Joos U, Buerger H (2005). HIF1-alpha overexpression indicates a good prognosis in early stage squamous cell carcinomas of the oral floor. BMC Cancer.

[42] Li C-X, Sun J-L, Gong Z-C, Lin Z-Q, Liu H (2016). Prognostic value of GLUT-1 expression in oral squamous cell carcinoma. Med (Baltim).

[43] Wang J, Ye C, Chen C, Xiong H, Xie B, Zhou J (2017). Glucose transporter GLUT1 expression and clinical outcome in solid tumors: a systematic review and meta-analysis. Oncotarget.

[44] Sitarz K, Czamara K, Szostek S, Kaczor A (2022). The impact of HPV infection on human glycogen and lipid metabolism – a review. Biochim et Biophys Acta (BBA) - Reviews Cancer.

[45] Fan R, Hou W-J, Zhao Y-J, Liu S-L, Qiu X-S, Wang E-H (2016). Overexpression of HPV16 E6/E7 mediated HIF-1α upregulation of GLUT1 expression in lung cancer cells. Tumour Biol.

[46] Fang J, Li X, Ma D, Liu X, Chen Y, Wang Y (2017). Prognostic significance of tumor infiltrating immune cells in oral squamous cell carcinoma. BMC Cancer.

[47] Höing B, Kanaan O, Altenhoff P, Petri R, Thangavelu K, Schlüter A (2018). Stromal versus tumoral inflammation differentially contribute to metastasis and poor survival in laryngeal squamous cell carcinoma. Oncotarget.

[48] Mendonça F, Teles AM, Nascimento MDDSB, Santos APAD, Lopes FF, Paiva A (2022). Human papillomavirus modulates Matrix metalloproteinases during carcinogenesis: clinical significance and role of viral oncoproteins. Vivo.

[49] Alkharsah KR (2018). VEGF upregulation in viral infections and its possible therapeutic implications. Int J Mol Sci.

[50] Jo S, Juhasz A, Zhang K, Ruel C, Loera S, Wilczynski SP (2009). Human papillomavirus infection as a prognostic factor in Oropharyngeal Squamous Cell Carcinomas Treated in a prospective phase II clinical trial. Anticancer Res.

[51] Karpathiou G, Stachowitz M-L, Dumollard JM, Gavid M, Froudarakis M, Prades JM (2019). Gene Expression Comparison between the Primary Tumor and its Lymph Node Metastasis in Head and Neck squamous cell carcinoma: a pilot study. Cancer Genomics Proteomics.

[52] Baruah P, Lee M, Wilson POG, Odutoye T, Williamson P, Hyde N (2015). Impact of p16 status on pro- and anti-angiogenesis factors in head and neck cancers. Br J Cancer.

[53] Troy JD, Weissfeld JL, Youk AO, Thomas S, Wang L, Grandis JR (2013). Expression of EGFR, VEGF, and NOTCH1 suggest differences in Tumor Angiogenesis in HPV-Positive and HPV-Negative Head and Neck squamous cell carcinoma. Head Neck Pathol.

[54] Kyzas PA, Stefanou D, Batistatou A, Agnantis NJ (2005). Prognostic significance of VEGF immunohistochemical expression and tumor angiogenesis in head and neck squamous cell carcinoma. J Cancer Res Clin Oncol.

[55] Tse GM, Chan AWH, Yu K-H, King AD, Wong K-T, Chen GG (2007). Strong immunohistochemical expression of vascular endothelial growth factor predicts overall survival in Head and Neck squamous cell carcinoma. Ann Surg Oncol.

