# Supplementary figures and images for: Aspartate-β-hydroxylase and hypoxia marker expression in head and neck carcinomas: implications for HPV-associated tumors
Source: Infect Agent Cancer. 2024 Jun 10;19:26. doi: 10.1186/s13027-024-00588-1 (PMC11163809; doi:10.1186/s13027-024-00588-1)

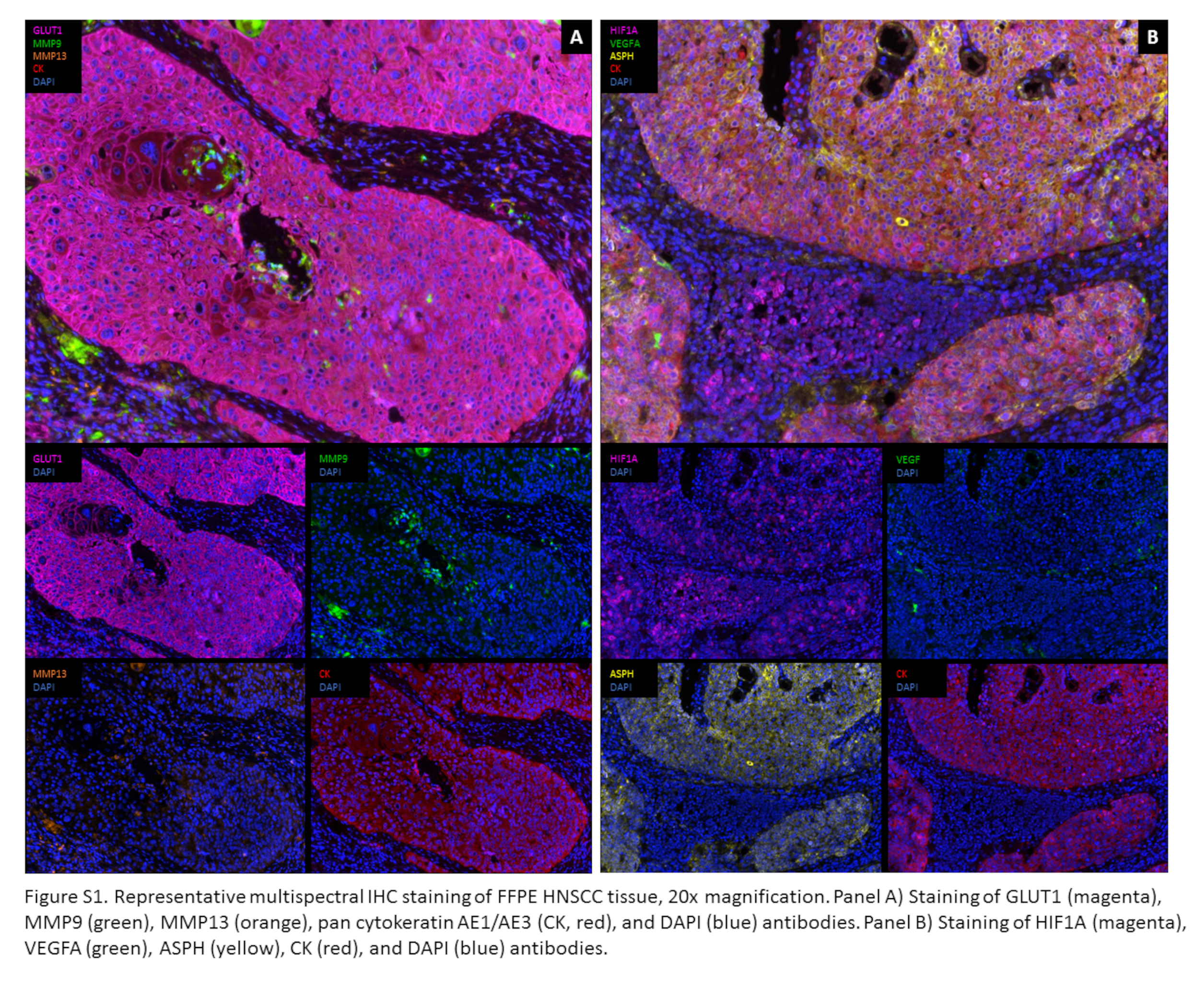

Supplement: Supplementary file 1 — Additional file 1. Figure S1. Representative multispectral IHC staining of FFPE HNSCC tissue, 20× magnification. (A) Panel A: Staining of GLUT1 (magenta), MMP9 (green), MMP13 (orange), pan cytokeratin AE1/AE3 (CK, red), and DAPI (blue) antibodies. (B) Panel B: Staining of HIF1A (magenta), VEGFA (green), ASPH (yellow), CK (red), and DAPI (blue) antibodies. [file 13027_2024_588_MOESM1_ESM.tiff]
