# Supplementary material for: Aspartate-β-hydroxylase and hypoxia marker expression in head and neck carcinomas: implications for HPV-associated tumors
Source: Infect Agent Cancer. 2024 Jun 10;19:26. doi: 10.1186/s13027-024-00588-1 (PMC11163809; doi:10.1186/s13027-024-00588-1)
Supplement: Supplementary file 2 — Additional file 2. Figure S2. ASPH and other hypoxia markers detected by the mIHC in the groups of HPV-positive (HPV+) and HPV-negative (HPV−) tumors (A), and in the parenchyma and stroma (B) of HNSCCs. The median value is indicated; the box borders show the upper and lower quartiles, the whiskers show the variability, and outliers are indicated. *p < 0.05, ** p < 0.01, *** p < 0.001, **** p < 0.0001. [file 13027_2024_588_MOESM2_ESM.docx]

**A**

**B**

**Figure S2**. ASPH and other hypoxia markers detected by the mIHC in the groups of HPV-positive (HPV+) and HPV-negative (HPV−) tumours (**A**), and in the parenchyma and stroma (**B**) of HNSCCs. The median value is indicated; the box borders show the upper and lower quartiles, the whiskers show the variability, and outliers are indicated. *p ˂0.05, ** p ˂0.01, *** p ˂0.001, **** p ˂0.0001
