# Supplementary material for: Aspartate-β-hydroxylase and hypoxia marker expression in head and neck carcinomas: implications for HPV-associated tumors
Source: Infect Agent Cancer. 2024 Jun 10;19:26. doi: 10.1186/s13027-024-00588-1 (PMC11163809; doi:10.1186/s13027-024-00588-1)
Supplement: Supplementary file 3 — Additional file 3. Table S1. Hazard ratio (HR) values for hypoxia markers influencing overall survival (OS) and disease-specific survival (DSS). [file 13027_2024_588_MOESM3_ESM.docx]

**Table S1**. Hazard ratio (HR) values for hypoxia markers influencing overall survival (OS) and disease-specific survival (DSS).

| **Model including HPV RNA status** | | | | | | | |
| --- | --- | --- | --- | --- | --- | --- | --- |
| OS | | | | DSS | | | |
|  | | | HR (p-value) |  | | | HR (p-value) |
| HPV RNA+ | | | 0.225 **(0.008)** | HPV RNA+ | | | 0.095 **(0.001)** |
| Increasing age | | | 1.065 **(0.027)** | Increasing age | | | 1.085 **(0.011)** |
| HIF1A** | | |  | MMP13** | | |  |
|  | | whole tumor | 0.818 (0.060) |  | whole tumor | | 1.109 (0.074) |
| **Models without HPV status inclusion** | | | | | | | |
| OS | | | | DSS | | | |
|  | | | HR (p-value) |  | | | HR (p-value) |
| Increasing age* | | | 1.068 **(0.016)** | Increasing age* | | | 1.087 **(0.015)** |
| GLUT1** | | |  | GLUT1** | | |  |
|  | whole tumor | | 0.802 **(< 0.0001)** |  | | whole tumor | 0.814 **(0.005)** |
|  | parenchyma | | 0.852 **(< 0.0001)** |  | | parenchyma | 0.862 **(0.009)** |
|  | stroma | | 0.814 **(0.007)** |  | | stroma | 0.842 **(0.032)** |
| OS | | | | DSS | | | |
|  |  | | HR (p-value) |  | | | HR (p-value) |
| Increasing age* | | | 1.060 **(0.037)** | Increasing age* | | | 1.076 **(0.026)** |
| HIF1A** | | |  | HIF1A** | | |  |
|  | whole tumor | | 0.729 **(0.003)** |  | | whole tumor | 0.773 **(0.021)** |
|  | parenchyma | | 0.829 **(0.008)** |  | |  |  |
|  | stroma | | 0.616 **(0.027)** |  | |  |  |

* HR for increasing age corresponds to the model calculated for the whole tumour area

** HR corresponds to a difference of 1000 positive cells/mm^2^
